# Discovery and Characterisation of Forward Line Formations at Centre Bounces in the Australian Football League

**DOI:** 10.3390/s23104891

**Published:** 2023-05-19

**Authors:** Daylon Seakins, Paul B. Gastin, Karl Jackson, Matthew Gloster, Aaron Brougham, David L. Carey

**Affiliations:** 1 Sport, Performance, and Nutrition Research Group, School of Allied Health, Human Services and Sport, La Trobe University, Melbourne, VIC 3083, Australiad.carey@latrobe.edu.au (D.L.C.); 2 Champion Data, Pty Ltd., Melbourne, VIC 3006, Australia

**Keywords:** Australian rules football, spatial formations, clustering, sport analytics, spatial data

## Abstract

The extent of player formation usage and the characteristics of player arrangements are not well understood in Australian football, unlike other team-based invasion sports. Using player location data from all centre bounces in the 2021 Australian Football League season; this study described the spatial characteristics and roles of players in the forward line. Summary metrics indicated that teams differed in how spread out their forward players were (deviation away from the goal-to-goal axis and convex hull area) but were similar with regard to the centroid of player locations. Cluster analysis, along with visual inspection of player densities, clearly showed the presence of different repeated structures or formations used by teams. Teams also differed in their choice of player role combinations in forward lines at centre bounces. New terminology was proposed to describe the characteristics of forward line formations used in professional Australian Football.

## 1. Introduction

**Figure 1:**
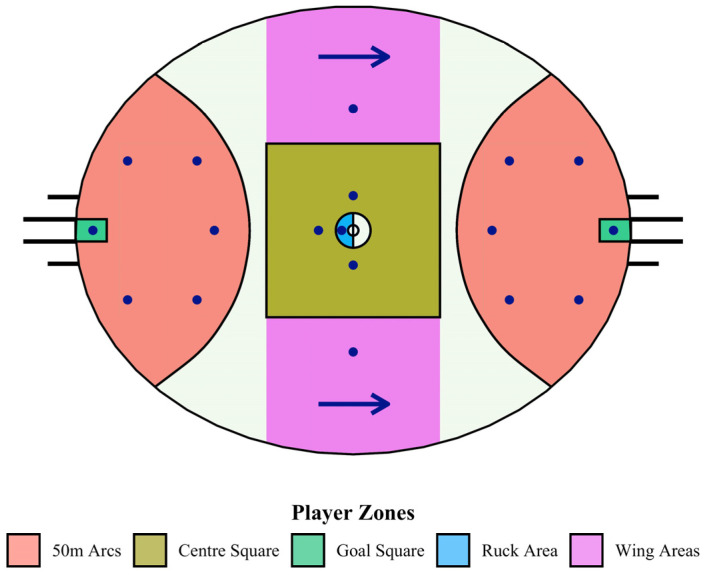
Diagram of the playing area in AF, showing player position requirements (for one team) at a centre bounce. Each dot represents a player that is free to position themselves anywhere within their coloured zone so long as the team meets the number requirement in each zone. The opposing team has the same requirements but is attacking in the opposing direction.

Australian football (AF) is a contact, team-based invasion sport played between two teams of 18 players (men’s version) on an oval-shaped field ranging from 155 to 175 m long and 116 to 145 m wide [1]. Teams compete to outscore the other by kicking an oval-shaped ball through two upright posts that form the “goals.” In professional and semi-professional AF, teams are required to start with six players within the 50 m arcs at either end of the playing ground (with at least one player in each goal square) when a centre bounce (CB) occurs (Figure 1) [2]. CBs are used at the start of each playing period to reset play after a goal is scored (26.7 times per match on average in 2021). At centre bounces, an umpire bounces the ball in the centre of the ground, and an equal number of players from each team compete to gain possession [3]. The team that gains possession of the ball will typically attempt to move it into their forward zone immediately, where the six forward line players are located. All players are free to move anywhere after the centre bounce is performed.

In team-based invasion sports opposing teams utilise player formations and structures in a tactical way to execute set strategies or game styles [4,5,6,7]. Formations and structures are an organised arrangement and positioning of players on a field relative to their teammates and opposition [4]. Teams that can organise themselves effectively into formations may be able to improve success rates by increasing their chances of scoring when in attack or stopping the opposing team from scoring when in defence. For example, it has been suggested that zone defences in AF were able to blunt the effectiveness of offensive strategies reliant on retaining ball possession [8] and that specific defensive formations in American football can reduce the offensive team’s ability to advance down the field [9]. Formations have been studied in soccer [10,11,12,13,14,15,16,17], American football [18,19,20,21], and hockey [22], reflecting the importance of formations in understanding how a sport is played. Additionally, it has been observed that soccer teams commonly switch the type of formation they use after losing a match [23], suggesting a belief among coaches that formations are a contributing factor to match outcomes. In Australian football (AF), there has been limited research investigating player positioning and the use of specific formations [24,25], leading to a gap in understanding, analysis, and commentary on the use of formations in the sport.

Soccer uses a widely known numbering system to describe the arrangement of lines of players in relation to each other, such as “343” or “4321,” to represent a count of players from defending to attacking [5]. American football uses colloquial names to describe set-play formations, such as “T-formation” or “Shotgun formation” [6]. In a similar sense to these other team-based invasion sports, there exists potential to create a set of terms for AF to describe formations that teams implement. Developing new terminology to describe formations in AF has the potential to improve communication and grow the understanding of the game and its tactics among fans, viewers, coaches, and analysts. It could provide a common reference for future research into team strategies and the interactions between different game styles. Additionally, the reporting of this novel data may inspire innovation regarding the use of formations in AF.

As a well-defined and frequently occurring phase of play, CBs present a promising starting point for the analysis of formations in AF. It could be expected that teams utilise their six forward line players in meaningful arrangements to best advantage their offensive play. It is not currently known if teams in professional men’s AF are using set forward-line formations that they repeat from match to match, how players are arranged in any formations used, what types of players teams are using in their forward lines, and what similarities or differences exist between the teams. The aims of this study were to discover and characterise player positioning and forward-line formations at CBs in AF. It was hypothesised that player location data would show evidence that teams were using structured formations at CBs in professional AF.

## 2. Materials and Methods

### 2.1. Data

In this descriptive study, data were captured from all the regular season (23 rounds) and finals (4 rounds) of the 2021 Australian Football League (AFL) season, consisting of 207 matches involving 18 professional teams (see Supplementary Table S1 for a list of team names and abbreviations). The AFL is the national and only full-time professional league for AF (men). The inclusion of data from every team and match provides a representative sample to detect and characterise player positioning strategies used in the league. Match event and player tracking data were provided by Champion Data Pty Ltd., the official data provider for the AFL. These data included the timestamp of each CB in each match (recorded manually to the nearest whole second by a data capture team familiar with the sport using a process containing multiple quality controls [26]), as well as 2D coordinates for each player on the field (recorded using wearable sensor devices). Player location data were captured using Catapult Vector devices sampling at 10 Hz [27]. Due to differences in the temporal resolution of these data sources, player locations at the time of centre bounces were calculated as the mean position over 11 samples centred on the timestamp of the CB. Player location data were transformed so that the origin point aligned with the centre of the goals, meaning the *y*-coordinate represented displacement away from the goal line toward the centre of the ground, and the *x*-coordinate was displacement away from the centre line of the ground. The six forward line players were assigned as the players with the smallest distance from the goals, and the closest player to the goal line was labelled as the goal square player. Player role labels (e.g., midfielder, key forward, or ruck) were also provided for each player by the commercial data provider. In cases where a player performed different roles throughout the season, their role was defined as the most frequent role played over the 2021 season. See Supplementary Table S2 for the list of possible roles and their descriptions.

Wearable player tracking data were collected using either a Local Positioning System (LPS) (one venue, 43/203 matches) or Global Positioning System (GPS) (all other venues, 160/203 matches) technologies [27]. LPS has been validated as accurate (errors typically <1 m) for player tracking purposes in sports [28,29]. However, the positional accuracy of GPS has been reported to be between 0.5 m [30] and 5 m [31] for sports activities. This, along with the low-resolution time stamps for centre bounces, meant that the player positions likely contained errors on the order of a few metres. The size of the playing field in AF and the typical spacing of forward players at centre bounces suggested it would still be possible to detect formations with the quality of location data available. To remove clearly erroneous data, if a team did not have six players within 55 m of the goal, then the CB was removed from the analysis. This was the only inclusion/exclusion criteria applied; otherwise, all centre bounce instances from the 2021 AFL season were included in the analysis. All data manipulation, visualisation, and analyses were conducted using the R statistical programming language [32].

### 2.2. Descriptive Analysis of Forward Player Positions at CBs

**Figure 2:**
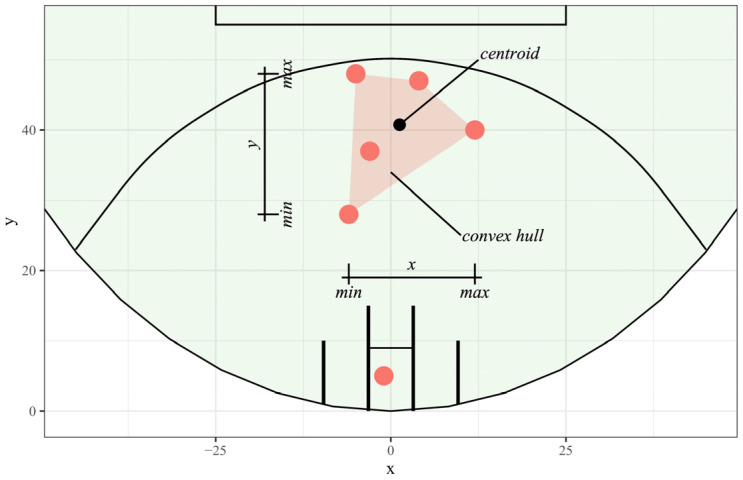
Example of forward line player positions at a centre bounce with annotations showing the descriptive metrics calculated.

To describe the spatial characteristics of the forward line, player positions at centre bounces were used to calculate the minimum and maximum *x*-coordinates and *y*-coordinates, the centroid, and the convex hull area (Figure 2). A convex hull is defined as the area within the outermost points of some geometry, and a centroid, in this instance, will be the mean coordinates of the overall formation [33]. These metrics provided a simple summary of how teams were positioning players and have been used in previous research [25,34]. The designated goal square player was omitted as this could be considered a “fixed position” that all teams were required to use with minimal flexibility. Team-based differences in these player position characteristics were assessed visually and with Kruskal–Wallis H tests, a non-parametric statistical test for group differences in a continuous variable [35].

### 2.3. Player Role Usage

The count and proportion that each player role (Supplementary Table S2) was used in a forward line formation by each team were calculated to describe and compare the types of players used by each team. Additionally, a four-digit naming convention (D-R-M-F) was developed to describe the combination of roles used at each CB, where D, R, M, and F represented the number of Defenders (D), Rucks (R), Midfielders, Mid-Forwards or Wings (M), and Forwards (F), respectively. Role combination frequencies were tallied for each team to compare their choice of player types to use in forward lines at centre bounces. Chi-squared tests were performed to test for team differences in the usage proportion of role combinations and the role of the GS player [36].

### 2.4. Forward Line Formation Discovery

Two methods were tested for spatial formation discovery. One relied on clustering frequent player locations and then describing formations using the cluster labels of the constituent players (frequent player location clustering method–Section 2.4.1).

**Figure 3:**
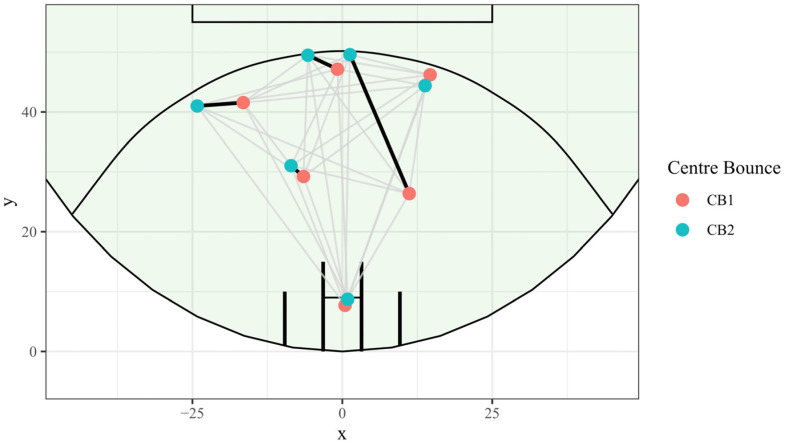
A comparison of two CBs, lines show all possible pairings of two sets of players, with optimal (minimum total distance) pairings shown in bold. The sum of the length of the bold lines was used to score the similarity between the two formations.

The other assigned the similarity between any two centre bounce formations as the solution to the minimum cost assignment problem [37] between the two sets of forward line players, where the cost of the assignment was the distance between the possible pairs of players (see Table 1 and Figure 3 for an example). The similarity scores were then used for hierarchical clustering to detect similar formation clusters in the distance matrix of all CBs (formation similarity clustering method–Section 2.4.2).

#### 2.4.1. Frequent Player Location Clustering Method

A total of 48,605 player locations at CBs were clustered using k-means clustering [38,39] to determine where forward line players most frequently positioned themselves when a CB occurred. Goal square (GS) players were excluded from the k-means analysis as they are required to be within a 6.4 × 9 m square, so they were considered to belong to a “fixed” GS cluster given the precision of data used. Following the cluster analysis, each CB formation could be described using the cluster label for each player, giving a five-letter code (six players minus one GS player). Players that were positioned within the GS but were not the required player (i.e., if the second nearest player to the goal was also within the goal square) were assigned to the GS cluster. The length of the player location vocabulary (the *k* in k-means clustering) was determined by calculating the average silhouette width [40] for different choices of *k* from 2 to 10 and by visually presenting the cluster centres to domain experts to assess their suitability for describing team formations. Domain experts considered the location and number of the returned cluster centres in the context of their prior knowledge of common forward line player locations used in the league. The chosen value of *k* was the value deemed suitable by experts that had the highest silhouette score.

#### 2.4.2. Formation Similarity Clustering Method

To discover frequently occurring spatial formations, the similarity between two sets of forward line players was calculated as the smallest total distance of pairings of players (solution to the minimum cost assignment problem) [41]. This approach was similar to previous studies in soccer [7,14,16,17,42,43,44] and hockey [22]. Formation similarity was calculated using the Hungarian algorithm [37,45], with the distance between two unique formations calculated as the total distance of pair-wise optimisations between players in each of the compared CBs. See Table 1 for comparisons between the six players in two unique CBs, the selected pairs and the total generated for the combination, and Figure 3 for how those optimal pairings appear visually.

Using all unique combinations of CBs, hierarchical clustering [39] was conducted on the distances of all the CB combinations. A “bottom-up” approach was used. The clustering method takes a distance matrix generated from the compared distances from all combinations of CBs, where each single CB was assigned a cluster to itself. From there, the two most similar clusters were combined iteratively until a single cluster was left [46]. Using the created line of clusters, they were “cut” at an appropriate point to select that number of clusters to represent these data. Consideration of average silhouette width and visual inspections of heatmaps based on different cluster prototypes and removal of clusters with few instances (<50) was undertaken in consultation with domain experts to determine where an appropriate cut-point was for the hierarchical clustering (in practice, the user of the method could use a different cut-point to suit their needs). Domain experts considered the spatial arrangement of each of the returned clusters in the context of their prior knowledge of how teams typically set up forward lines in the AFL.

#### 2.4.3. Team Comparisons

To compare the variability of forward line formations used by different teams, the entropy (Equation (1)) [47] of each team’s formation usage was calculated. Entropy was calculated using all formations observed in the league rather than every possible one, as these formations were considered plausible enough for a team to use.

(1) $$H^{T}=-\sum_{i=1}^{n}p_{i}^{T}\log p_{i}^{T},$$

where *n* is the number of formations observed across the whole league, and $p_{i}^{T}$ is the proportion that team *T* used formation *i*. A team that used only one formation would have zero entropy, and a team using all observed formations in equal proportion would have the maximum entropy.

## 3. Results

Forward line player positions were examined in 9721 CBs from 203 matches after 1082 CBs (10.02%) were removed for not having enough players within 55 m of the goal, indicating device tracking or time synchronisation errors above the accepted threshold (see Supplementary Table S3 for rejection rates at each venue). The sample included 523 unique players observed in at least one CB forward line (median 29 per team, range 22–34). The median number of CBs per team was 535.5 (range 468–645).

**Figure 4:**
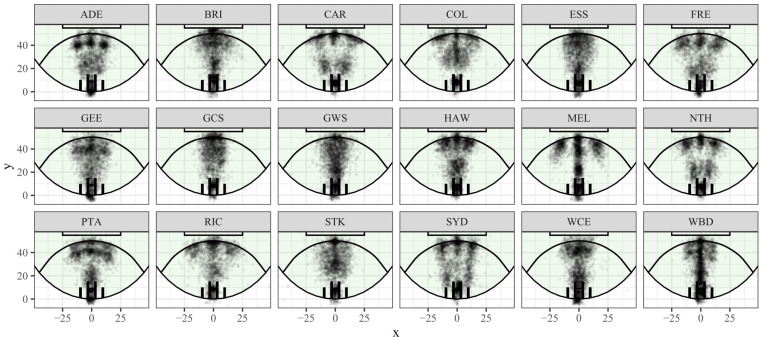
Distributions of players per team at each CB, darker areas indicate locations where teams more frequently had players positioned.

Figure 4 shows the distribution of forward line players per team at all CBs in these data. Several teams could be seen as having distinct darker areas, giving a clear indication of some organised underlying structure used by teams. MEL had a distinct “T” shape to their distribution, many teams had three distinct darker areas spaced along the 50 m arc (noticeably in ADE, FRE, NTH, and SYD distributions), and MEL and the WBD had general straight-line shapes from the GS to the top of the 50 m arc. This visualisation shows players from all CBs by team overlayed on each other, so different “types” of layouts could be overlapped and could be the case for some of the noisier distributions. This reinforced the necessity for formation discovery to identify these underlying formations.

### 3.1. Descriptive Analysis of Forward Player Positions at CBs

**Figure 5:**
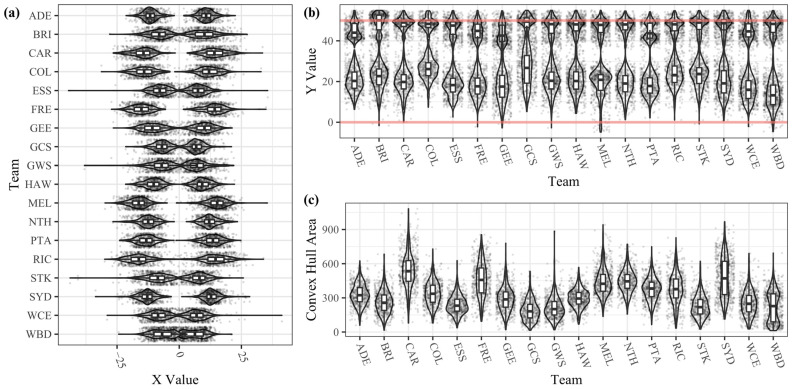
(**a**) Distributions of the *x*-axis position of the leftmost and rightmost player at each centre bounce for each team. (**b**) Distributions of the *y*-axis position of the highest and deepest non-GS player at each centre bounce for each team. The higher red line indicates the 50 m arc, and the lower red line indicates the goal line. (**c**) Convex hull area of forward line formations by team, excluding the GS player.

The minimum and maximum *x* and *y*-coordinates, the centroid, and the convex hull area were calculated for all forward lines. Figure 5a is a double box plot graph with violins and points showing the distribution of the minimum *x*-coordinate (leftmost player) and the maximum *x*-coordinate (rightmost player). All teams appeared mostly symmetrical; however, some differences were apparent in the typical widths of the distributions, with some teams narrower (WBD, ESS, STK, GCS, GWS) or wider (MEL, FRE, RIC) than others. All teams’ distributions of both metrics appeared unimodal, bar the Western Bulldogs (WBD).

Figure 5b shows the minimum and maximum *y*-coordinates of the teams in the league. The upper box plot shows the distribution of the maximum *y*-coordinate (highest player), while the lower box plot shows the minimum *y*-coordinate distribution (deepest non-GS player). There was little variation in the highest player between teams, with most typically opting to have a player at or within a few metres of the 50 m line (the maximum allowable distance from the goal). There was more variation in the deepest non-GS player between teams, with GCS and COL appearing to favour higher minimum *y*-coordinates and WBD often positioning a player closer to the goal line.

Figure 5c shows the convex hull area of forward line formations (excluding the GS player). There appeared to be a distinguishable variety amongst teams’ usage here. Several distributions appeared bimodal, such as HAW and WBD, which may suggest the teams use more than one distinct formation. CAR and SYD had higher areas, or more spread-out players, while others favoured a tighter arrangement (STK, GCS, GWS, WBD).

**Figure 6:**
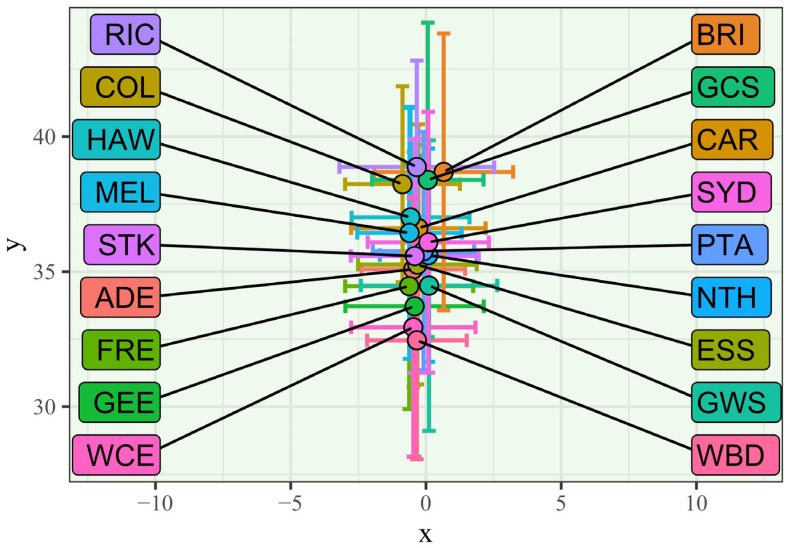
Mean centroids and standard deviations of forward line formations of AFL teams at CBs.

Figure 6 shows the centroids of the teams in the league. The arrangement of teams was close together, with minimal difference in the *x*-coordinate. There was variation between the depth (*y*-axis) of the teams’ centroids, but they were still all within 6.4 m of each other. Kruskal–Wallis Tests indicated that inter-team differences in all descriptive formation metrics (the minimum and maximum *x*-coordinate and *y*-coordinates, the centroid, and the convex hull area) were statistically significant (*p* < 0.001). However, visual inspection of Figure 4, Figure 5 and Figure 6 suggests that the centroid locations and typical highest player were not meaningfully different between teams.

### 3.2. Player Role Usage Comparisons

**Figure 7:**
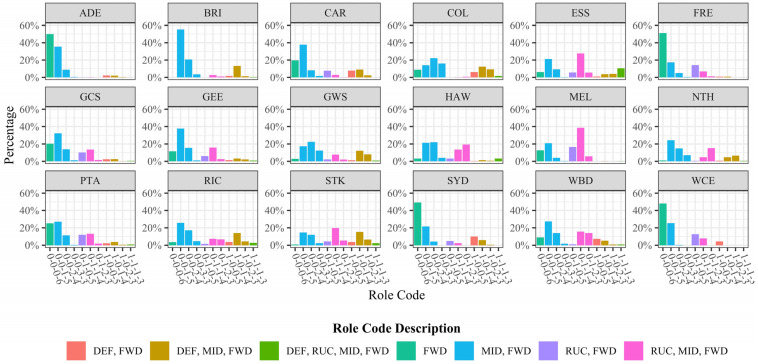
Usage proportions for each of the top 11 player role combinations (D-R-M-F).

There were 27 unique role usage combinations (D-R-M-F) present amongst teams in the league, with teams exhibiting different preferences for usage proportions (χ^2^ = 8149.1, *p* < 0.001). Eleven combinations were used more than 100 times and made up 95.2% of the CBs in the dataset. The relative frequency that each team used each of the 11 most common combinations is shown in Figure 7. Visually, it can be observed that teams are not using the same combinations at the same relative frequencies and that some teams or groups of teams are exhibiting recurring role combinations. Groups appear to exist, including teams that use mostly only Forwards (ADE, FRE, SYD, and WCE), or high Ruck, and Midfielder and Forward utilisation (ESS, HAW, and STK).

Supplementary Figure S1 shows the breakdown of roles used in the goal square, showing differences in team tendencies (χ^2^ = 2898.7, *p* < 0.001). There was a higher proportion of Key Forwards than General Forwards in goal square usage compared to the rest of the formation role uses. The Ruck role was also utilised in the goal square a notable proportion of the time. COL used a Mid-Forward in their goal square position the most of any other team at 22.53% of their uses.

### 3.3. Forward Line Formation Discovery

#### 3.3.1. Frequent Player Location Clustering

**Figure 8:**
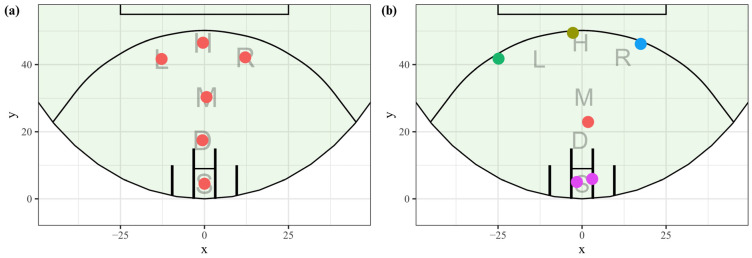
(**a**) Labels and centroids of clustered frequent player locations: all players within the forward 50 m arc. (**b**) An example formation showing how players within the forward line are assigned to a frequent player location cluster to generate the formation label, in this instance, LHMRS. One S (GS) player is assumed.

The maximum average silhouette width from the k-means clustering was at *k* = 2, with minimal differences in score between *k* = 3 and *k* = 8. It was deemed, in consultation with domain experts, that *k* ≤ 4 was not a high enough resolution to describe how teams were positioning their players properly and that *k* = 5 was the best trade-off between complexity and representation. The average silhouette width returned from *k* = 5 was 0.376. The returned cluster centroids (Figure 8a) appeared reasonably symmetrical. A goal square cluster was added manually after player location data were clustered and centred within the 6.4 × 9 m goal square. The clusters were assigned a letter label; L for left; H for high; M for middle; D for deep; R for right. This system allowed for the creation of formation codes, with more popular formations still able to be assigned more descriptive, colloquial names. The manual goal square cluster was assigned S for short. See Figure 8a for the location and labels of the cluster centroids. Figure 8b is an example formation showing the underlying centroid locations and how players within a formation would be assigned to a cluster to generate a formation label for the CB formation (*LHMRS* in this instance).

**Figure 9:**
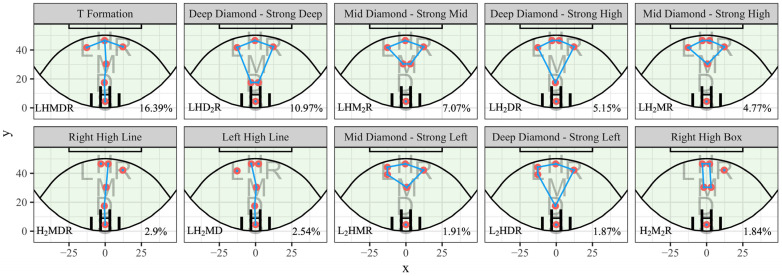
The top 10 most frequently used formations (frequent player location clustering method), with accompanying formation labels, rates, and colloquial names.

There were 151 unique formations observed across all teams in the league. With 65 formations making up 95% of them. While 25 had 100 or more uses. The top 10 most frequently used formations can be seen in Figure 9. The most popular formations were also assigned colloquial names. Names were used to reflect shapes or patterns that the used combination of clusters resembled.

Table 2 shows the entropy of teams’ formation usage (frequent player location clustering method). NTH had the lowest entropy, indicating their formation usage was the least variable. Their most frequent formation was *LHD_2_R* which was used 33.81% of the time from 34 unique formations. GWS had the highest entropy suggesting their formation usage had the most uncertainty; their most used formation was *H_2_M_2_D* and was used just 6.23% of the time from 87 unique formations. Supplementary Table S4 shows each team’s top five formations used.

#### 3.3.2. Formation Similarity Clustering

**Figure 10:**
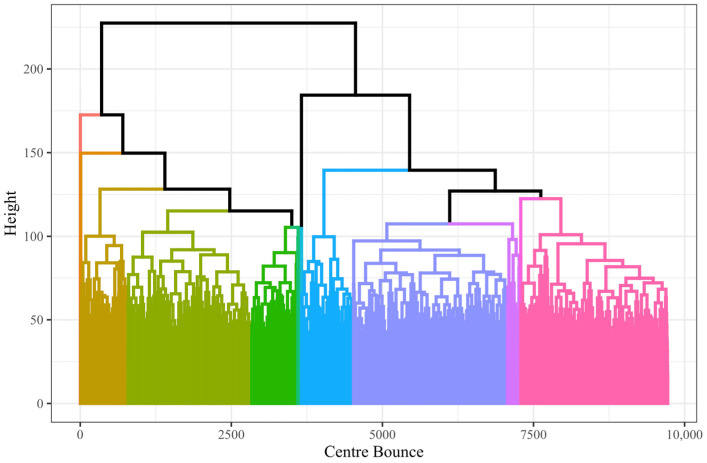
Dendrogram of CBs with colours showing cluster labels when cut at a height returning 13 clusters (some of the small clusters are not visible).

**Figure 11:**
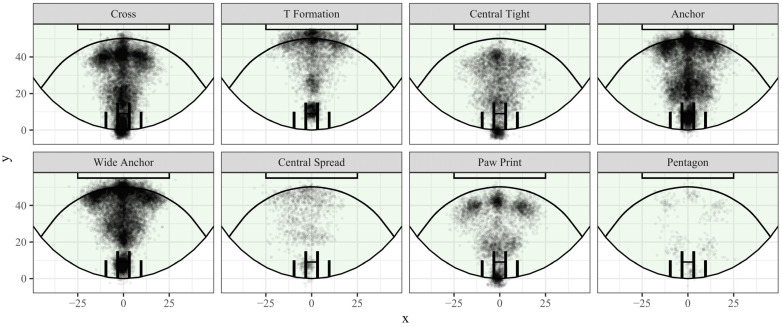
The distribution and density of players in each of the eight primary clusters (formation similarity clustering) and proposed colloquial names. Darker areas indicate locations where teams more frequently had players positioned.

In the formation similarity clustering approach, the similarity scores for all possible comparisons (47,244,060) between the 9721 CBs were calculated, and hierarchical clustering was applied to the distance matrix. Figure 10 is the resulting dendrogram showing the possible cut heights for generating clusters for the hierarchical clustering of formations’ similarity. Consideration of the average silhouette width produced and visual inspection of the heatmaps based on different cluster count prototypes (with the removal of clusters with few instances, <50) was undertaken in consultation with domain experts to determine where an appropriate cut was made. It was determined that 13 clusters produced the best balance of complexity and representation. A total of 13 clusters returned an average silhouette width of 0.015. Five of the thirteen clusters had fewer than one hundred instances and were omitted in favour of eight clusters (capturing 99.8% of observations) for representing forward line formations. Figure 11 shows the distribution and density of players in those eight clusters.

Table 3 shows the entropy of teams’ formation usage (formation similarity clustering method). The entropy across the league was similar, with a range of 0.45. WBD had the lowest entropy, which suggests their formation usage through this method had the smallest uncertainty in predicting future usage. GEE had the highest entropy, suggesting they were the least predictable.

**Figure 12:**
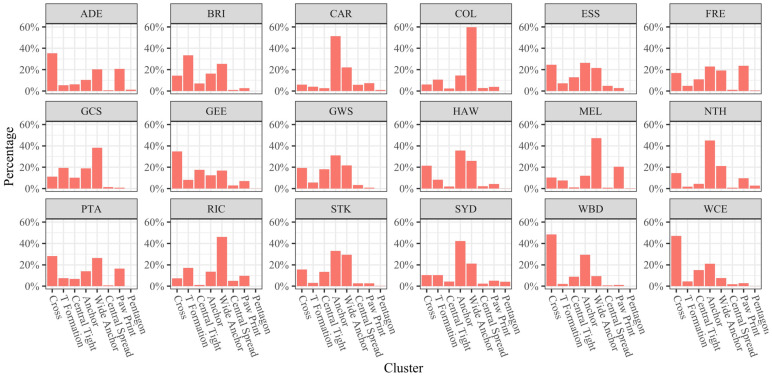
Cluster usage rates by team (formation similarity clusters).

Figure 12 shows the formation similarity cluster usage by team. The usage of clusters appeared different between teams; however, some groups appeared to have similar utilisations. ESS, FRE, GWS, and STK appeared similar, so to were NTH and SYD, and ADE and PTA.

## 4. Discussion

The aim of this study was to investigate the forward line formations used by AFL teams at centre bounces. The results presented are the first published description of how teams at the professional level are positioning their players during this phase of the game. The visualization in Figure 4 clearly showed that player positioning strategies varied between teams, and repeated structures or formations, were present in these data, similar to other sports [10,11,12,13,14,15,16,17,18,19,20,21,22]. Teams differed in their choice of player-type combinations in their forward lines at CBs (Figure 7). The exploratory cluster analyses performed were the first attempt at detecting and characterizing specific player formations. Names and naming systems were proposed to begin to address the gap in formation understanding identified in AF.

### 4.1. Player Positioning at CBs

Kruskal–Wallis test statistics and visual inspection of CB metrics (minimum and maximum *x* and *y*-coordinates, the centroid, and the convex hull area) suggested that teams employed meaningfully different forward line formations (Figure 5 and Figure 6, Kruskal–Wallis *p* < 0.001 for all metrics). Possible explanations include different player compositions between teams, different beliefs from coaches about the optimal way to position players to receive an incoming ball, and different potential strategies for moving the ball into the forward zone after a CB (e.g., favouring a longer kick to a deeper location vs a shorter pass higher into the forward zone). Additionally, since opposition tracking data are not readily available in AFL and these data have not been published before, there has been less opportunity for teams to converge towards a homogenous set of strategies.

The maximum *y*-coordinate, or highest player up the ground (Figure 5b), did not show much variation between teams (i.e., most teams had at least one player beyond 40 m and near the 50 m arc). At a CB, the ball is always located in the centre of the ground, so the highest point in the forward zone would be the closest point to the ball (see Figure 1). A player in this position would therefore have the shortest distance to travel to the ball location to assist midfield players if the ball were to spill loose or if the opposition gained possession.

The differing distributions of the convex hull area metric (Figure 5c) and the *x*-axis spread (Figure 5a) indicated that teams differed in how much they tended to spread out forward-line players. However, in general, teams appeared to favour positioning players in a symmetrical way on the *x*-axis (goal-to-goal axis). Given the symmetry of the playing field and the difficulty in predicting or controlling the outcome of centre bounces [3], it would make sense for teams to exhibit no preference for one side of the ground. Symmetry about the goal-to-goal axis has also been observed in soccer formations [11,16]. Network analysis in AF suggests that winning probability is improved when more players are involved in a team’s passing network (i.e., a well-connected network) [48], with opportunities for scoring available from both sides of the field desirable. In support of this, a defensive team formation that is wider than it is long also seems preferred to defend inside 50 entries [24].

### 4.2. Comparing Player Role Usage Rates

There were clear differences in the league with regard to their player role-usage combinations (Figure 7, χ^2^ = 8149.1, *p* < 0.001), providing further evidence that meaningful differences exist and that teams are employing intentional strategies. Different player role usage rates have been used in American football [49], basketball [50], and cricket [51], and this study provides evidence that AF teams are also using this approach. The most common combinations of player types were all forwards, forwards with midfielders, and forwards with rucks (Figure 7), and teams exhibited clearly different usage rates for these combinations. Factors driving the differences could be different team compositions or individual player abilities (e.g., a ruck player who has shown the ability to operate effectively as a forward player or a team that selects two rucks to play in a match and must rotate one of them to another position at CBs). Alternatively, a team strategy or tactic may influence the structure, such as putting a defender in the forward line to counteract a specific opposition player or a midfielder starting in the forward zone and then moving or swapping with another player after the CB in an attempt to disrupt the defence. Further investigation into the movements of players after CBs would be required to understand the intentions of teams.

Key Forwards and rucks were the most popular player role used in the goal square (Supplementary Figure S1), aligning with traditional playing roles of a tall player being the deepest forward. Team differences were also apparent in the choice of player role used in the goal square (χ^2^ = 2898.7, *p* < 0.001).

### 4.3. Detecting Forward Line Formations

Formations were able to be detected by both methods used, consistent with previous studies [7,13,14,22,43] where formations were able to be detected using a variety of approaches. Between the frequent player location clustering and the formation similarity clustering, similar patterns were apparent across both sets of results. Formations were symmetric about the goal-to-goal axis and included at least one player near the top of the 50 m arc but varied with respect to the relative position of other players, amount of spread, and arrangement of depth.

Arrangement of players into what could be labelled a ‘cross’, ‘T’, ‘anchor’, or ‘arrow’ formation was popular among multiple teams and was detected by both methods (Figure 4, Figure 9 and Figure 11). Similarly, a ‘diamond,’ ‘fan,’ or ‘paw-print’ type formation was evident in the results. Variations of these motifs (wide, narrow, deep, or high) allowed for a richer set of terms to characterise what teams were doing with player positions at CBs.

All teams had entropy values clearly smaller than 5.02 (a random choice entropy), showing that team strategies were not random. This may just suggest that some teams subscribe to setting their forward line formation stringently or that potentially teams with a wider variety of formations are using similar formations, but these are registering as different within the analysis. To account for this in future studies or if this terminology was used in practice, groups of similar formations could be established to reflect how teams may be using them.

The purpose of this research was exploratory and descriptive, so the results of both methods are presented for comparison and as a reference for future investigations into formations in AF. Since the frequent player location clustering approach worked off individual player locations, and the formation similar approach computed similarity between whole arrangements of players, comparing the methods under clustering evaluation metrics was not possible.

### 4.4. Applications

Data presented in this study and the proposed terminology, similar to that used in other team-based invasion sports [5,22,52], have the potential to assist in the understanding and innovation of strategies and tactics used by AF teams. This could aid future AF research, enhance the knowledge of coaches at elite and improve fan and media discussions and understanding of the game. Terminology to identify and characterise formations could also potentially lead to enhanced opposition analysis at the professional level. Beyond the description of formation characteristics, the colloquial names assigned to the more popular formations could enhance the reach of discussion, commentary, and wider-fan conversation around forward-line formations at CBs.

This study analysed data from an entire season of professional men’s AF (all teams). The findings are likely relevant for other elite AF leagues that use the 6-6-6 rule [2], such as the top-tier state leagues and top junior competitions in Australia. Women’s AF has two fewer players on the field, and a 5-6-5 rule at centre bounces, meaning that forward line formations may be different to those used in the men’s league. The discovery and descriptive methods used in this study would be suitable to profile the use of forward formations in women’s AF. The generalisability of findings to lower-level competitions and younger age groups is less clear, with fewer coaches, fewer datasets available, and different player skill profiles suggesting the use of formations may be less prominent.

### 4.5. Limitations and Future Directions

The accuracy of player position data available was a limitation of the study, particularly at venues where GPS had to be used to locate players [30,31]. Approximately 10% of all observed CBs had to be removed from analyses due to clear positional errors (players >55 m from the goal), and imprecision in player positions may have obscured some features of the detected formations. Improvements to the quality of tracking data in AF may lead to further insights into formation usage.

Two methods for formation detection were tested in this study; however, other approaches, such as Gaussian mixture models [53] and non-negative matrix factorization [4], have been used for similar problems in other sports and may be appropriate for future research in AF.

Data used in the study consisted of matches from the 2021 season. The rule restricting teams to six players in the forward zone at CBs was introduced at the conclusion of the 2018 season; it would be of interest and potential value to include 2019–2020 and 2022 data in this work to possibly identify any changes in strategy over that period. Analysing temporal trends in style and monitoring team sports gameplay are important to understand the evolution of a sport and the emergent trends in coaching [8].

Analysis of player movement immediately after CBs could be incorporated into future studies. How players move once the formation “breaks” is important contextual information. Players that stand near or on the 50 m arc frequently jog or sprint towards the centre circle to act as an extra midfielder once the CB occurs. Players sometimes rotate around to a wing to allow a defender to move up to the midfield quickly, with the existing wing rotating to defence. With this incorporation, further insights into team game styles and behaviour could be integrated with the newly created formation vocabulary. As an example, spatiotemporal behavioural analyses derived from similar player tracking data have recently described both individual and team tactical behaviour (e.g., player movement and positioning, spacing, synchrony, unpredictability, dispersive coordination) in AFL football [54].

This study did not explore how formation usage influenced outcomes of play (such as scoring or attaining a high-quality shot on goal) or if teams used different formations in response to different match situations (e.g., after being scored against or when protecting a lead in a close match). Future research could aim to explore the motivations for different formation usage and their impact on match outcomes.

## 5. Conclusions

This study is the first descriptive analysis of forward-line player positions at CBs in Australian football. These data showed clear evidence of structured formation usage that differed between teams. Teams also showed different tendencies in their choice of player-type combinations in the forward line at CBs. Cluster analyses showed that formations were typically symmetrical around the goal-to-goal axis and included at least one player near the top of the 50 m arc. However, the positioning of other players differed with respect to spread, relative position, and depth. There were also differences in the predictability of teams, with some strongly favouring a small number of formations and using them repeatedly and others having a more uniform distribution of formation usage. Future research could enhance the understanding of formations in AF by examining player positioning and movement patterns during other facets of gameplay and by testing for relationships between formation usage and performance outcomes.

## Figures and Tables

**Table 1:** Example comparison of formations using the Hungarian algorithm. Pairings of players selected from the matrix to give the minimum total distance (total = 44.18).

	CB2: Player 1	CB2: Player 2	CB2: Player 3	CB2: Player 4	CB2: Player 5	CB2: Player 6
**CB1: Player 1**	7.69 *	13.38	19.52	30.47	13.22	37.17
**CB1: Player 2**	24.18	5.45 *	3.21	14.88	17.87	38.45
**CB1: Player 3**	39.18	20.64	13.79	1.98 *	27.71	39.91
**CB1: Player 4**	21.28	20.27	21.79	25.34	2.75 *	21.77
**CB1: Player 5**	38.26	28.62	25.22 *	18.21	20.25	20.42
**CB1: Player 6**	41.42	42.21	41.86	39.03	24.99	1.09 *

* Optimal pairing (minimum total distance).

**Table 2:** The entropy of each team’s formation (frequent player location clustering method) usage.

Team	Entropy	Team	Entropy
NTH	1.45	HAW	2.19
MEL	1.58	STK	2.51
RIC	1.81	WBD	2.64
ADE	1.9	ESS	2.64
PTA	1.93	BRI	2.69
FRE	1.95	GCS	2.74
CAR	2.09	WCE	2.77
SYD	2.13	GEE	2.91
COL	2.19	GWS	3.02

**Table 3:** The entropy of each team’s formation (formation similarity clustering method) usage.

Team	Entropy	Team	Entropy
WBD	1.31	STK	1.6
COL	1.31	BRI	1.62
CAR	1.47	GWS	1.64
MEL	1.48	SYD	1.67
WCE	1.48	ADE	1.67
NTH	1.54	PTA	1.69
RIC	1.55	ESS	1.72
HAW	1.56	GEE	1.74
GCS	1.58	FRE	1.76

## Data Availability

The data are not publicly available due to commercial sensitivity.

## Supplementary Materials

The following supporting information can be downloaded at: https://www.mdpi.com/article/10.3390/s23104891/s1, Figure S1: Count of player roles used in the goal square of forward line formations by team; Table S1: AFL team names and abbreviations; Table S2: Abbreviated AF player role names and respective “zones” [55]; Table S3: Centre bounce rejection rates for different venues. Centre bounces were rejected if there were not six players within 55 m of the goal, indicating player position errors above the accepted threshold; Table S4: Top five formations (frequent player location clustering method) per team.
