# Supplementary material for: Discovery and Characterisation of Forward Line Formations at Centre Bounces in the Australian Football League
Source: Sensors (Basel). 2023 May 19;23(10):4891. doi: 10.3390/s23104891 (PMC10222966; doi:10.3390/s23104891)
Supplement: Supplementary file 1 [file sensors-23-04891-s001.zip › sensors-2318482-supplementary.pdf]

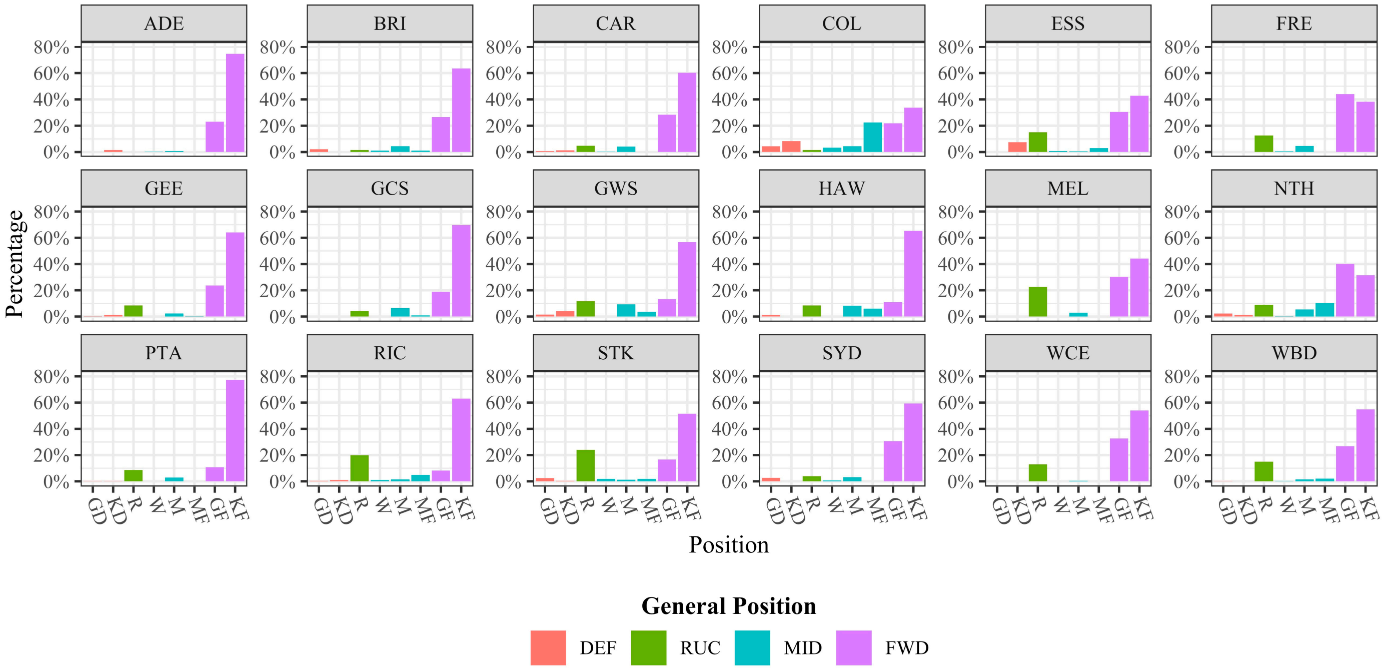

Supplementary Figure S1. Count of player roles used in the goal square of forward line formations by team.

Supplementary Table S1. AFL team names and abbreviations.

Team	Abbreviation	Team	Abbreviation
Adelaide	ADE	Hawthorn	HAW
Brisbane Lions	BRI	Melbourne	MEL
Carlton	CAR	North Melbourne	NTH
Collingwood	COL	Port Adelaide	PTA
Essendon	ESS	Richmond	RIC
Fremantle	FRE	St Kilda	STK
Geelong	GEE	Sydney Swans	SYD
Gold Coast SUNS	GCS	West Coast Eagles	WCE
Greater Western Sydney GIANTS	GWS	Western Bulldogs	WBD

Supplementary Table S2. Abbreviated AF player role names and respective "zones" [55].

Position	Abbreviation	Description
General Defender	GD	Plays on the general forward and usually sets up play and wins more of the ball than a key defender.
Key Defender	KD	A tall or mid-sized defender who plays on the opposition's key forward
Wing	W	A player who aligns on the sides of the centre square at a centre bounces and follows the play predominantly on a single wing of the field.
Ruck	R	A player who contests at least 35% of hit-outs for their team.
Midfielder	M	A player who predominantly starts inside the centre square at centre bounces and follows the ball all across the field.
Mid-Forward	MF	A player who spends at least 35% of their time as both a midfielder/wing or forward.
General Forward	GF	A small/medium player that predominantly plays in the forward half of the ground.

Key Forward	KF	A taller player who can take contested marks and is usually one of the focal points inside 50.

Supplementary Table S3. Centre bounce rejection rates for different venues. Centre bounces were rejected if there were not six players within 55 m of the goal, indicating player position errors above the accepted threshold.

Venue Name	Total CBs	N rejected	Rejection Rate (%)
Sydney Cricket Ground (SCG)	589	119	20.2
Gabba	630	109	17.3
GIANTS Stadium	360	58	16.11
GMHBA Stadium	558	78	13.98
Metricon Stadium	653	72	11.03
Marvel Stadium	2405	232	9.65
Adelaide Oval	1184	109	9.21
Melbourne Cricket Ground (MCG)	2088	169	8.09
Optus Stadium	1326	104	7.84
Blundstone Arena	296	21	7.09
Cazaly's Stadium	40	2	5
University of Tasmania Stadium	416	7	1.68
Mars Stadium	144	2	1.39
Manuka Oval	114	0	0

Supplementary Table S4. Top five formations (frequent player location clustering method) per team.

Team	Formation	Percentage Used	Team	Formation	Percentage Used	Team	Formation	Percentage Used
	LHMDR	28.14%		LHMDR	13.93%		LHMDR	24.73%
	LHD_2R	19.32%		LHD_2R	7.82%		LH_2DR	16.18%
ADE	LHM_2R	13.13%	GEE	LH_2DR	5.15%	PTA	$LHDR_2$	11.82%
	LH_2MR	4.69%		LHM_2R	4.01%		LHD_2R	8.18%
	$LHMD_2$	3.19%		LMD_2R	3.44%		L_2HDR	7.27%
	LH_2MR	9.38%		H_3M_2	6.41%		LH_2DR	20.3%
	LH_2DR	8.85%		H_3MR	5.61%		LH_2MR	19.44%
BRI	H_3MR	7.43%	GCS	LH_2MR	5.41%	RIC	LHMDR	12.82%
	H_2MDR	4.96%		LH_2M_2	5.01%		LHM_2R	11.75%
	LHMDR	4.96%		H_2M_2R	4.81%		LHD_2R	4.91%
	LHD_2R	26.33%		H_2M_2D	6.23%		LHM_2R	10.08%
	LHMDR	25%		HM_2DR	5.69%		H_2M_3	7.86%
CAR	LHM_2R	9.85%	GWS	LHMDR	4.63%	STK	H_2M_2D	5.85%
	L_2HDR	4.17%		HM_2D_2	4.27%		LH_2M_2	5.65%
	L_2HMR	3.98%		HMD_2R	3.91%		LHMDR	5.24%
	LHM_2R	20.99%		LHMDR	26.94%		LHD_2R	29%
	LHMDR	11.88%		LHM_2R	15.03%		LHMDR	17.44%
COL	LM_3R	7.52%	HAW	LHD_2R	9.67%	SYD	LHM_2R	8.36%
	LH_2MR	6.93%		H_2MDR	6.74%		LH_2MR	7.47%
	L_2HMR	4.95%		LH_2MD	5.87%		LMD_2R	3.91%
	H_2MDR	10.97%		LHMDR	40.83%		LHD_2R	12.52%
	LH_2MD	8.18%		LH_2DR	18.02%		LHMDR	8.71%
ESS	LHMDR	7.62%	MEL	LH_2MR	10.25%	WCE	H_2D_2R	6.72%
	HM_2DR	5.58%		LHM_2R	8.43%		$LHMD_2$	6.35%
	H_3MD	5.58%		LHDRS	4.96%		LH_2MD	5.99%

FRE	LHD_2R	24.18%	NTH	LHD_2R	33.81%	WBD	LHD_2R	10.85%
	LHMDR	19.34%		LHMDR	27.94%		LHMDR	9.77%
	$LHDR_2$	6.19%		LHM_2R	9.31%		H_2M_2D	7.91%
	L_2HDR	5.22%		L_2HMR	2.63%		HM_2D_2	7.6%
	LMD_2R	4.64%		LHDRS	2.43%		H_2MD_2	7.13%
